# An exploratory, randomised, placebo-controlled, 14 day trial of the soluble guanylate cyclase stimulator praliciguat in participants with type 2 diabetes and hypertension

**DOI:** 10.1007/s00125-019-05062-x

**Published:** 2019-12-19

**Authors:** John P. Hanrahan, Jelena P. Seferovic, James D. Wakefield, Phebe J. Wilson, Jennifer G. Chickering, Joon Jung, Kenneth E. Carlson, Daniel P. Zimmer, Andrew L. Frelinger, Alan D. Michelson, Linda Morrow, Michael Hall, Mark G. Currie, G. Todd Milne, Albert T. Profy

**Affiliations:** 1 Cyclerion Therapeutics, Inc., 301 Binney Street, Cambridge, MA 02142 USA; 2 Center for Platelet Research Studies, Dana-Farber/Boston Children’s Cancer and Blood Disorders Center, Boston, MA USA; 3 ProSciento, Inc., Chula Vista, CA USA; 4 Ironwood Pharmaceuticals, Inc., Cambridge, MA USA

**Keywords:** BP, Cyclic guanosine monophosphate, Endothelial function, Hyperlipidaemia, Hypertension, Insulin resistance, Soluble guanylate cyclase stimulator, Type 2 diabetes

## Abstract

**Aims/hypothesis:**

Praliciguat (IW-1973), a soluble guanylate cyclase stimulator, amplifies nitric oxide signalling. This exploratory trial investigated the safety, tolerability, pharmacokinetic profile and pharmacodynamic effects of praliciguat in individuals with type 2 diabetes and hypertension.

**Methods:**

This Phase IIA, double-blind, placebo-controlled trial investigated praliciguat in 26 participants with type 2 diabetes and hypertension on stable glucose- and BP-lowering therapies. Participants were randomly allocated in a 3:5:5 ratio to three groups: placebo (*n* = 6), praliciguat 40 mg once daily for days 1–14 (*n* = 10), or praliciguat 20 mg twice daily for days 1–7 then 40 mg once daily for days 8–14 (*n* = 10). Assessments were made in clinic and included treatment-emergent adverse events, pharmacokinetics, metabolic variables, 24 h BP and heart rate, platelet function, reactive hyperaemia index (RHI) and plasma biomarkers. Participants, the sponsor, the investigator and clinic study staff (except designated pharmacy personnel) were blinded to group assignment.

**Results:**

Participants treated for 14 days with praliciguat had least-square mean change-from-baseline differences vs placebo (95% CI) of −0.7 (−1.8, 0.4) mmol/l for fasting plasma glucose, −0.7 (−1.1, −0.2) mmol/l for total cholesterol, −0.5 (−1.0, −0.1) mmol/l for LDL-cholesterol, −23 (−56, 9) for HOMA-IR in those not being treated with insulin, and −5 (−10, 1) mmHg and 3 (−1, 6) beats/min for average 24 h mean arterial pressure and heart rate, respectively. Apart from one serious adverse event (SAE; upper gastrointestinal haemorrhage), praliciguat was well tolerated. Praliciguat did not affect platelet function or RHI. Among exploratory biomarkers, plasma levels of asymmetric dimethylarginine decreased in praliciguat vs placebo recipients.

**Conclusions/interpretation:**

In participants with type 2 diabetes and hypertension on standard therapies, over 14 days praliciguat was well tolerated, except for a single SAE, and showed positive trends in metabolic and BP variables. These results support further clinical investigation of praliciguat.

**Trial registration:**

ClinicalTrials.gov NCT03091920.

**Funding:**

This trial was funded by Cyclerion Therapeutics.

**Electronic supplementary material:**

The online version of this article (10.1007/s00125-019-05062-x) contains peer-reviewed but unedited supplementary material, which is available to authorised users.

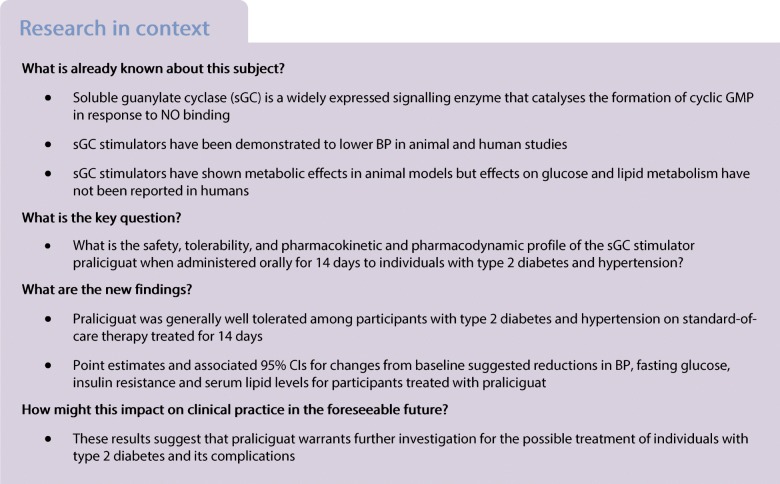



## Introduction

The global prevalence of type 2 diabetes has increased rapidly over recent decades. In 2015, the Centers for Disease Control and Prevention reported that diabetes affected 9.4% of the US population, 90–95% of whom had type 2 diabetes, and was the seventh leading cause of death [1]. Type 2 diabetes and its complications represent a substantial burden and cost to patients, providers, the healthcare system and society at large in the developed world.

Type 2 diabetes is associated with endothelial dysfunction, reduced NO bioavailability and impaired NO signalling [2, 3]. Endothelial dysfunction and disrupted NO signalling have been implicated in the pathophysiology of macro- and microvascular complications of diabetes [4]. Soluble guanylate cyclase (sGC), a central enzyme in the NO signalling pathway, catalyses the conversion of GTP to cyclic GMP (cGMP) in response to NO binding. NO–sGC–cGMP signalling influences an array of physiological processes including vascular tone, inflammation, fibrosis and metabolism.

sGC stimulators are small-molecule allosteric agonists that sensitise sGC to endogenous NO and increase cGMP production. They have shown activity in preclinical models of cardiovascular, renal, metabolic and fibrotic disorders [5–7]. These observations provide a rationale for clinical investigation of sGC stimulators for the treatment of diabetes and its complications, such as nephropathy, neuropathy and retinopathy.

Praliciguat (IW-1973), an investigational sGC stimulator, decreased BP and protected against end-organ damage in non-clinical disease models relevant to diabetes and hypertension [8]. In addition, it demonstrated positive metabolic effects in a diet-induced obesity mouse model [9] and reduced proteinuria and fasting blood glucose in the ZSF1 rat model of diabetic nephropathy [6]. In healthy adults, plasma cGMP increased and haemodynamic effects were observed when praliciguat 15–40 mg was administered daily for up to 21 days [10]. We conducted a Phase IIA exploratory trial to evaluate the safety, tolerability, pharmacokinetic and pharmacodynamic profile of praliciguat in individuals with type 2 diabetes and hypertension.

## Methods

### Study design and participants

This was a randomised, double-blind, placebo-controlled, in-clinic, exploratory Phase IIA trial conducted at a single centre (ProSciento, Chula Vista, CA, USA). Eligible participants were aged 30–75 years, had type 2 diabetes and hypertension for at least 6 months, and a BMI of 20–40 kg/m^2^. They were required to be on a stable medication regimen for a minimum of 28 days prior to randomisation, including either an ACE inhibitor (ACEi) or an angiotensin receptor blocking agent (ARB), and at least one glucose-lowering agent. Additional BP- and glucose-lowering treatments were permitted. All participants were required to have baseline HbA_1c_ ≤ 91 mmol/mol (≤10.5%), fasting plasma glucose ≤13.3 mmol/l, systolic BP (SBP) 120–160 mmHg and diastolic BP (DBP) 70–100 mmHg. Hepatic impairment, bleeding history/disorder, cancer, severe end-organ morbidity and the use of any investigational drug within 30 days were exclusionary. Pregnant and lactating women were excluded, and stringent birth control was required during the trial.

Three to twenty-eight days after a screening visit, 26 eligible participants were randomised in a 5:5:3 ratio to receive the following treatment: praliciguat 40 mg once daily on days 1–14 (*n* = 10); praliciguat 20 mg twice daily on days 1–7 then 40 mg once daily on days 8–14 (*n* = 10); or placebo (*n* = 6). The computer-generated blocked randomisation schedule was prepared by an independent statistician, not otherwise associated with the study, using a block size of 13. Two praliciguat treatment regimens were tested to evaluate potential for different tolerability profiles. Praliciguat was provided as a 5 mg oral tablet and placebo tablets matched praliciguat tablets in appearance. All participants were dosed with eight tablets (either praliciguat or placebo) twice daily throughout the 14 day treatment period to maintain the blind. Participants were discharged from the clinic on day 15 and returned for follow-up visits approximately 21 and 42 days after dose initiation.

The study was conducted in accordance with the Declaration of Helsinki and International Conference on Harmonisation (ICH) Guidelines for Good Clinical Practice. The study protocol, amendments and consent form were approved by the Institutional Review Board. All participants provided written informed consent before participation.

### Pharmacokinetics

Plasma concentrations of praliciguat were measured using a validated LC–tandem MS bioanalytical method, as previously described [10]. Non-compartmental pharmacokinetic calculations, using actual sampling times relative to dosing, were performed with Phoenix WinNonlin Version 6.4 (Certara LP, Princeton, NJ, USA) and included the maximum observed plasma concentration (C_max_), time of C_max_ (T_max_), AUC for the plasma concentration–time curve, apparent volume of distribution during the terminal elimination phase (V_z_/F) and the effective *t*_½_ [11] (electronic supplementary material [ESM] Table 1).

### Safety and pharmacodynamic assessments

Treatment-emergent adverse events (TEAEs), vital signs, serum chemistry, haematology, coagulation and urinalysis (including urine creatinine) were assessed. Twenty-four-hour ambulatory BP monitoring (ABPM) with measurements at intervals of 30 min was conducted at baseline and on days 1, 7 and 14. To estimate insulin resistance, HOMA-IR was calculated from fasting plasma glucose and serum insulin levels [12]. The eGFR was calculated using the Chronic Kidney Disease Epidemiology Collaboration (CKD-EPI) creatinine equation [13]. Other assessments included reactive hyperaemia index (RHI) and augmentation index corrected to a heart rate of 75 beats/min (bpm) (AI_75_), measured by digital plethysmography (EndoPAT, Itamar Medical, Caesarea, Israel). Inflammatory, vascular injury and lipoprotein biomarkers (ESM Table 2) were also assessed.

Platelet function was assessed by VerifyNow (both aspirin and P2Y_12_ assays; Accumetrics, San Diego, CA, USA) and by flow cytometric measurement of both platelet surface activated glycoprotein IIb-IIIa and platelet surface P-selectin expression, as described previously [14]*.* These analyses were conducted with whole blood and platelet-rich plasma under both unstimulated and stimulated conditions with two platelet activators: ADP and thrombin receptor activating protein (TRAP) at two concentrations (Boston Children’s Hospital Center for Platelet Research Studies).

### Statistical analysis

The planned sample size of 26 participants was determined outside of statistical considerations but was considered adequate to achieve the exploratory objectives of the trial. Data from all participants were used for all analyses. Analyses of change-from-baseline pharmacodynamic data were performed using ANCOVA with treatment as a fixed effect and baseline as a covariate. Least squares (LS) mean for each treatment and LS mean differences from placebo, along with the associated 95% CIs obtained from the model were rounded. Outcomes examined in this trial were not powered for inferential testing: analyses were descriptive and focused on estimation. All results should thus be considered exploratory and hypothesis-generating. All statistical analyses were performed using SAS Version 9.1 (SAS Institute, Cary, NC, USA).

## Results

A total of 26 participants were randomised to one of the two praliciguat 40 mg daily groups (20 mg twice daily for 7 days, then 40 mg once daily for 7 days; 40 mg once daily for 14 days; *n* = 10 both groups) or the placebo group (*n* = 6). Of the 26 participants randomised, 25 (96%) completed the study as planned. One person treated with praliciguat discontinued dosing due to an adverse event (described below). Baseline characteristics were generally similar, with no clinically significant differences, when comparing the praliciguat- and placebo-treated participants and the praliciguat twice daily, praliciguat once daily and placebo-treated participants (Table 1). No participant changed their BP-lowering regimen during the treatment phase of the trial.

**Table 1:** Participant characteristics at baseline

Characteristic	Placebo (*n* = 6)	Praliciguat 40 mg daily		
		Twice daily / once daily^a^ (*n* = 10)	Once daily / once daily^b^ (*n* = 10)	Overall (*n* = 20)
Age, years	61 ± 6	61 ± 8	63 ± 7	62 ± 7
Male sex, *n* (%)	2 (33)	6 (60)	5 (50)	11 (55)
Race/ethnicity, *n* (%)				
White	6 (100)	7 (70)	7 (70)	14 (70)
Asian	0	1 (10)	2 (20)	3 (15)
Black or African-American	0	2 (20)	1 (10)	3 (15)
Hispanic or Latino	5 (83)	2 (20)	3 (30)	5 (25)
Weight, kg	87 ± 20	92 ± 19	82 ± 20	87 ± 20
BMI, kg/m^2^	32 ± 3	33 ± 4	31 ± 5	32 ± 5
Haemodynamic variables				
Systolic BP, mmHg	132 ± 8	126 ± 11	129 ± 6	128 ± 9
Diastolic BP, mmHg	77 ± 9	72 ± 9	72 ± 6	72 ± 7
MAP, mmHg	96 ± 8	90 ± 7	91 ± 6	91 ± 6
Heart rate, bpm	75 ± 14	73 ± 11	75 ± 11	74 ± 11
Metabolic variables				
Plasma glucose, mmol/l	7.9 ± 1.8	9.0 ± 2.8	8.2 ± 1.9	8.6 ± 2.4
HbA_1c_, mmol/mol	60.1 ± 14.3	62.2 ± 11.8	63.7 ± 11.8	63.0 ± 11.5
HbA_1c_, %	7.7 ± 1.3	7.8 ± 1.1	8.0 ± 1.1	7.9 ± 1.1
HOMA-IR^c^	7.4 ± 5.2	6.6 ± 3.4	7.2 ± 5.7	7.0 ± .7
Serum insulin, pmol/l^c^	146 ± 107	126 ± 53	123 ± 91	125 ± 80
Total cholesterol, mmol/l	4.0 ± 1.3	3.9 ± 0.7	4.3 ± 0.9	4.1 ± 0.8
LDL-cholesterol, mmol/l	1.9 ± 1.1	2.2 ± 0.6	2.1 ± 0.9	2.2 ± 0.8
HDL-cholesterol, mmol/l	1.2 ± 0.3	1.1 ± 0.4	1.2 ± 0.3	1.1 ± 0.4
Triacylglycerol, mmol/l	1.7 ± 0.7	1.3 ± 0.6	2.3 ± 1.4	1.8 ± 1.2
eGFR, ml min^−1^ [1.73 m]^−2^	103 ± 8	89 ± 20	80 ± 20	85 ± 20
Medication, *n* (%)				
Metformin	6 (100)	7 (70)	9 (90)	16 (80)
Sulfonylurea	2 (33)	2 (20)	4 (40)	6 (30)
Dipeptidyl peptidase-4 inhibitor	0 (0)	0 (0)	1 (10)	1 (5)
Insulin	2 (33)	5 (50)	3 (30)	8 (40)
Statin	3 (50)	7 (70)	8 (80)	15 (75)
ACEi	2 (33)	7 (70)	6 (60)	13 (65)
ARB	4 (67)	3 (30)	4 (40)	7 (35)
*β*-Adrenergic blocker	1 (17)	1 (10)	1 (10)	2 (10)
Calcium channel blocker	1 (17)	5 (50)	1 (10)	6 (30)
Diuretic	1 (17)	3 (30)	3 (30)	6 (30)
Aspirin	3 (50)	6 (60)	6 (60)	12 (60)

Data are mean ± SD, unless otherwise indicated

^a^20 mg twice daily for 7 days, then 40 mg once daily for 7 days

^b^40 mg once daily for 14 days

^c^Participants not on insulin (*n* = 4 for placebo group; *n* = 5 for praliciguat twice daily/once daily group; *n* = 7 for praliciguat once daily/once daily group)

Praliciguat was rapidly absorbed, with a median T_max_ of 1–3 h, and the two regimens produced similar dose-normalised C_max_ (group mean: 5.84 ng ml^−1^ mg^−1^ for twice daily dosing; 6.05 ng ml^−1^ mg^−1^ for once daily dosing) after 7 days of dosing. After 14 days of dosing, the V_z_/F was large (overall mean 4680 l), and the overall mean effective *t*_½_ was 39.7 h. Detailed pharmacokinetic variables are presented in ESM Table 1.

**Fig. 1:**
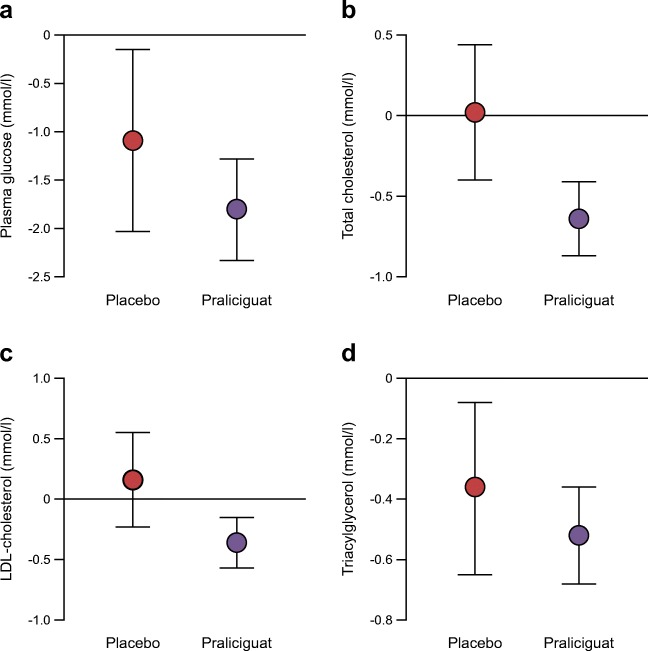
Changes in metabolic variables from baseline to week 2. Data are presented as LS mean change from baseline with 95% CIs. LS mean differences (95% CI) between praliciguat (*n* = 19) and placebo-treated (*n* = 6) participants were as follows: (**a**) plasma glucose, −0.7 (−1.8, 0.4) mmol/l; (**b**) total cholesterol, −0.7 (−1.1, −0.2) mmol/l; (**c**) LDL-cholesterol, −0.5 (−1.0, −0.1) mmol/l; and (**d**) triacylglycerol, −0.2 (−0.5, 0.2) mmol/l

Pharmacodynamic results after 14 days of treatment were similar for the two praliciguat regimens (twice daily/once daily vs once daily/once daily) and are presented combined. LS mean changes from baseline to day 14, as well as LS mean differences from placebo in metabolic variables, are presented in Fig. 1. Decreases from baseline in fasting plasma glucose were seen in both praliciguat and placebo groups, and the point estimate and associated 95% CI for the difference suggests decreases were greater in praliciguat-treated patients (LS mean difference −0.7 [95% CI −1.8, 0.4] mmol/l). Changes from baseline in HbA_1c_ were small and similar in the praliciguat- and placebo-treated groups (LS mean [95% CI] −0.3 [−0.5, −0.2]% (−3.6 [−5.2, −2.0] mmol/mol) and −0.3 [−0.6, −0.0]% (−3.4 [−6.3, −0.5 mmol/mol]), respectively; LS mean difference from placebo [95% CI] 0 [−0.3, 0.3]% (−0.2 [−3.5, 3.2] mmol/mol).

Among the subset of 16 participants taking oral glucose-lowering agents but not insulin (*n* = 12 praliciguat, *n* = 4 placebo), the point estimate and 95% CIs suggest the decrease from baseline in fasting plasma glucose was greater in participants treated with praliciguat than in those receiving placebo (LS mean [95% CI] −1.6 [−2.1, −1.1] mmol/l and −0.5 [−1.3, 0.3], respectively; LS mean difference from placebo [95% CI] −1.1 [−2.0, −0.1] mmol/l). In this subgroup, decreases following praliciguat vs placebo treatment were also suggested for HOMA-IR (LS mean [95% CI] −36 [−53, −19] and −13 [−40, 15], respectively; LS mean difference from placebo [95% CI] −23 [−56, 9]). In addition, LS mean change [95% CI] from baseline in fasting serum insulin for praliciguat- and placebo-treated participants was −36 [−54, −19] pmol/l and −23 [−54, 7] pmol/l, respectively; LS mean difference from placebo [95% CI] −13 [−49, 22] pmol/l.

Reductions from baseline in both total cholesterol and LDL-cholesterol were observed in praliciguat-treated participants (LS mean [95% CI] −0.6 [−0.9, −0.4] mmol/l and −0.4 [−0.6, −0.1] mmol/l, respectively), while no reduction was observed in placebo-treated participants (LS mean [95% CI] 0.0 [−0.4, 0.4] mmol/l and 0.2 [−0.2, 0.5] mmol/l, respectively). LS mean difference (95% CI) from placebo for total cholesterol and LDL-cholesterol was −0.7 (−1.1, −0.2) mmol/l and −0.5 (−1.0, −0.1) mmol/l, respectively. Decreases from baseline in serum triacylglycerol were observed in both praliciguat- and placebo-treated participants, with the point estimate and 95% CI suggesting greater reductions in those treated with praliciguat (LS mean difference [95% CI] −0.2 [−0.5, 0.2] mmol/l). Changes from baseline in HDL-cholesterol levels were small and similar between praliciguat- and placebo-treated participants (LS mean [95% CI] −0.05 [−0.14, 0.03] mmol/l and −0.01 [−0.16, 0.14] mmol/l, respectively; LS mean difference [95% CI] for praliciguat vs placebo −0.04 [−0.21, 0.13] mmol/l). Point estimates and 95% CIs for changes from baseline in apolipoprotein B also suggested greater decreases in praliciguat-treated participants than in placebo-treated participants (LS mean difference [95% CI] −2.0 × 10^−4^ [−6.0 × 10^−4^, 1.0 × 10^−4^] mmol/l; see ESM Table 2 for full results of apolipoprotein B and other lipoprotein biomarkers). Point estimates and 95% CIs for change from baseline in lipid levels for the subset of 18 participants on concomitant statin therapy (15 praliciguat, 3 placebo) also suggested greater declines in praliciguat-treated vs placebo-treated participants: total cholesterol (LS mean difference [95% CI] −0.4 [−1.1, 0.3] mmol/l) and LDL-cholesterol (LS mean difference [95% CI] −0.4 [−1.1, 0.2] mmol/l).

Neither body weight nor BMI changed from baseline over the treatment period for either praliciguat-treated or placebo-treated participants, with no differences between the groups (LS mean difference [95% CI] 0.0 [−1.1, 1.1] kg and 0.0 [−0.4, 0.4] kg/m^2^, respectively).

**Fig. 2:**
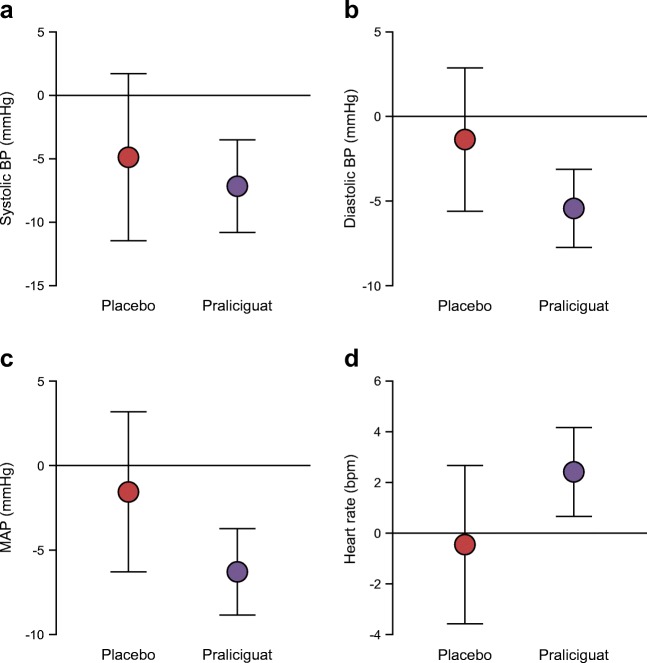
Changes in haemodynamic variables from baseline to week 2 measured by 24 h ABPM. Data are presented as LS mean change from baseline with 95% CIs. LS mean differences (95% CI) between praliciguat-treated (*n* = 19) and placebo-treated (*n* = 6) participants were as follows: (**a**) systolic BP, −2 (−10, 5) mmHg; (**b**) diastolic BP, −4 (−9, 1) mmHg; (**c**) MAP, −5 (−10, 1) mmHg; and (**d**) heart rate, 3 (−1, 6) bpm

Figure 2 presents changes from baseline in average 24 h haemodynamic variables as measured by ABPM. At day 14, point estimates and CIs suggest greater reductions from baseline for praliciguat-treated participants vs placebo-treated participants in average 24 h measurements (LS mean difference [95% CI] of −2 [−10, 5] mmHg for SBP, −4 [−9, 1] mmHg for DBP and −5 [−10, 1] mmHg for mean arterial pressure [MAP]). Point estimates and 95% CI for change in average 24 h heart rate suggested greater increases in praliciguat-treated participants than in placebo-treated participants (LS mean difference 3 [95% CI −1, 6] bpm).

In post hoc analyses of subgroups stratified by median baseline BP, praliciguat-treated participants with baseline MAP >92 mmHg had a greater LS mean difference from placebo-treated participants (−14 [−23, −5] mmHg) than praliciguat-treated participants with baseline MAP ≤92 mmHg (LS mean difference 2 [95% CI −4, 8] mmHg). A similar pattern was seen for SBP and DBP.

At the pre-dose measurement on day 13, change from baseline in RHI was similar in praliciguat-treated and placebo-treated participants (LS mean [95% CI] 0.09 [−0.17, 0.34] and 0.28 [−0.17, 0.74], respectively; LS mean difference [95% CI] −0.20 [−0.73, 0.34]). AI_75_ declined from baseline in both praliciguat-treated and placebo-treated participants (LS mean [95% CI] −4.8 [−9.0, −0.6] and −0.99 [−9.02, 7.04], respectively; LS mean difference [95% CI] −3.9 [−13.2, 5.5]).

Changes from baseline and LS mean differences for biomarkers of inflammation and vascular injury are presented in ESM Table 2. Point estimates and associated 95% CIs suggest that, compared with placebo-treated participants, praliciguat-treated participants had a greater decline in asymmetric dimethylarginine (ADMA; LS mean difference −10.7 [95% CI −18.7, −2.6] ng/ml) and a greater increase in l-arginine/ADMA ratio (LS mean difference 22.2 [95% CI 7.5, 36.8]). Changes in other plasma biomarkers were similar between the two treatment groups.

There were no values or changes in values indicative of a praliciguat effect on platelet function as assessed by VerifyNow. This instrument measures platelet function using whole blood samples. For the VerifyNow P2Y_12_ assay, values below 180 P2Y_12_ reaction units (PRU), a measure of ADP-induced platelet aggregation assessed by an increase in light transmission, suggest evidence of a P2Y_12_ inhibitor effect [15]. At both baseline and day 14, all participants had results above 180 PRU. For the VerifyNow aspirin test, values below 550 aspirin reaction units (ARU) indicate an aspirin-like inhibitory effect on arachidonic acid-induced platelet aggregation [16]. At baseline, all participants had values consistent with their reported aspirin use or no use, except for one person with reported concomitant aspirin use who had a value above 550 ARU. At day 14, three praliciguat-treated participants with reported concomitant aspirin use had values above 550 ARU, one praliciguat-treated participant with no report of concomitant aspirin use had a value below 550 ARU, and all other participants had values consistent with their reported aspirin use. In addition, praliciguat-treated participants did not show meaningful changes from baseline or differences from placebo in analyses of platelet surface activated glycoprotein IIb-IIIa and platelet surface P-selectin expression under unstimulated and stimulated conditions in both whole blood and platelet-rich plasma (data not shown).

Fourteen (70%) of the 20 participants treated with praliciguat experienced at least one TEAE compared with five of six participants (83%) treated with placebo (Table 2). One person taking praliciguat had a serious adverse event (SAE) and discontinued praliciguat after 11 days of treatment. All other participants completed 14 days of treatment.

**Table 2:** Treatment-emergent adverse events

MedDRA preferred term	Placebo (*n* = 6)	Praliciguat 40 mg		
		Twice daily / once daily^a^ (*n* = 10)	Once daily / once daily^b^ (*n* = 10)	Overall (*n* = 20)
Any TEAE	5 (83)	6 (60)	8 (80)	14 (70)
Headache	2 (33)	2 (20)	3 (30)	5 (25)
Hypoglycaemia	2 (33)	2 (20)	3 (30)	5 (25)
Nausea	0	2 (20)	3 (30)	5 (25)
Diarrhoea	1 (17)	0	3 (30)	3 (15)
Abdominal pain	0	0	2 (20)	2 (10)
Dyspepsia	0	1 (10)	1 (10)	2 (10)
Injection site haemorrhage	3 (50)	0	1 (10)	1 (5)
Cough	0	0	1 (10)	1 (5)
Dry throat	0	0	1 (10)	1 (5)
Oropharyngeal pain	0	0	1 (10)	1 (5)
Alopecia	0	0	1 (10)	1 (5)
Gastrointestinal sounds abnormal	0	0	1 (10)	1 (5)
Costochondritis	0	0	1 (10)	1 (5)
Paronychia	0	0	1 (10)	1 (5)
Dizziness	1 (17)	1 (10)	0	1 (5)
Anaemia	0	1 (10)	0	1 (5)
Eye irritation	0	1 (10)	0	1 (5)
Dry mouth	0	1 (10)	0	1 (5)
Eructation	0	1 (10)	0	1 (5)
Gastroesophageal reflux disease	0	1 (10)	0	1 (5)
Oesophagitis	0	1 (10)	0	1 (5)
Upper gastrointestinal haemorrhage	0	1 (10)	0	1 (5)
Vomiting	0	1 (10)	0	1 (5)
Muscle spasms	0	1 (10)	0	1 (5)
Pain in extremity	0	1 (10)	0	1 (5)
Injection site injury	0	1 (10)	0	1 (5)
Dermatitis contact	0	1 (10)	0	1 (5)
Pseudohypoglycaemia	1 (17)	0	0	0
Limb discomfort	1 (17)	0	0	0
Tremor	1 (17)	0	0	0
Nephrolithiasis	1 (17)	0	0	0
Nocturia	1 (17)	0	0	0

Data are presented as *n* (%)

^a^20 mg twice daily for 7 days, then 40 mg once daily for 7 days

^b^40 mg once daily for 14 days

MedDRA, Medical Dictionary for Regulatory Activities

All TEAEs other than the single SAE were characterised as mild. The most common TEAEs were headache (5/20 [25%] praliciguat, 2/6 [33%] placebo), nausea (5/20 [25%] praliciguat, 0/6 placebo) and hypoglycaemia (5/20 [25%] praliciguat, 2/6 [33%] placebo). Other TEAEs reported in two or more praliciguat-treated participants were diarrhoea (3/20 [15%] praliciguat, 1/6 [17%] placebo), abdominal pain (2/20 [10%] praliciguat, 0 placebo) and dyspepsia (2/20 [10%] praliciguat, 0/6 placebo).

Headache occurred in approximately the same proportion of praliciguat- and placebo-treated participants. Of the 3 of 10 (33%) participants in the once daily/once daily praliciguat group who reported headache, all had onset in the first week of dosing. Of the 2 of 10 (20%) participants in the twice daily/once daily praliciguat group who reported headache, one had onset in the first week and one in the second week. Of the 2 of 6 (33%) placebo-treated participants who reported headache, onset was reported by one in the first week and one in the second week.

Gastrointestinal TEAEs were reported in 9/20 (45%) of praliciguat-treated participants, compared with 1/6 (17%) of placebo-treated participants. Five of the praliciguat-treated participants reported multiple gastrointestinal events; all others reported a single event. Of the 5 of 10 (50%) participants in the once daily/once daily praliciguat group who had at least one gastrointestinal adverse event, two had event onsets in both weeks, one had onset in the first week only, and two had onset in the second week. Of the 4 of 10 (40%) participants in the twice daily/once daily praliciguat group, two had onsets in both weeks, one had onset only in the first week, and one only in the second week. The participant in the placebo group who experienced gastrointestinal adverse effects had onset in the second week.

All five praliciguat-treated participants who experienced hypoglycaemia were among the eight taking concomitant insulin, as was one of two placebo-treated participants who reported this adverse event.

Only two participants, one on the 40 mg once daily dose of praliciguat and one on placebo, had an adverse event that could be consistent with low BP (dizziness).

A single SAE occurred in a 59-year-old man, who developed a spontaneous upper gastrointestinal haemorrhage after 11 days of dosing and was hospitalised for 1 day. Treatment was discontinued for the remainder of the trial. Prior to this episode, the participant’s concomitant medications included captopril and insulin, and he had no history of gastrointestinal bleeding. Endoscopic evaluation identified a hiatal hernia with ulcerative oesophagitis but no source of active bleeding. The participant did not have further gastrointestinal bleeding and recovered uneventfully.

## Discussion

This exploratory Phase IIA trial found that participants with type 2 diabetes and hypertension treated for 2 weeks with the sGC stimulator praliciguat had trends toward improvement in metabolic and haemodynamic outcomes. Decreases in fasting plasma glucose, insulin, HOMA-IR and serum lipids were seen in both praliciguat- and placebo-treated participants but the LS mean differences between the groups consistently suggested greater changes in praliciguat-treated participants. Glucose control as assessed by HbA_1c_ was unchanged, as expected in a trial of such short (14 days) treatment duration. Meaningful assessment of glucose control would need to be explored in longer clinical investigations.

Reduction in fasting plasma glucose in praliciguat-treated participants was suggested in those on stable regimens for glycaemic control, including the subset of participants who were not receiving concomitant insulin treatment. Praliciguat-related decline in mean lipid levels was also observed in the subset of participants on concurrent statin therapy. These subgroup results suggest that praliciguat may provide additional glucose- and lipid-lowering effects on top of the current standard of care. However, these results should be interpreted with caution, as the subgroups were small.

Participants treated with praliciguat had a greater mean decrease from baseline in average 24 h BP compared with placebo. All participants in this trial were already on a stable antihypertensive regimen that included an ACEi or an ARB, suggesting an additional BP-lowering effect of praliciguat treatment. Reductions in BP are consistent with the known vasodilatory effects of modulators of the NO–sGC–cGMP signalling pathway [17–19]. The decreases in BP were seen predominantly in participants having baseline values above the median level for the cohort. Greater BP-lowering effects in participants with diabetes and inadequate BP control would be advantageous, especially if accompanied by few hypotension-associated adverse events, as in this trial.

Haemodynamic effects of sGC stimulation are known to be mediated through vascular smooth muscle but may be affected by endothelial NO release. In this trial, the effects of praliciguat on endothelial function were evaluated by peripheral arterial tonometry and by plasma biomarkers. Praliciguat showed no clear effect on RHI [20], a non-invasive tonometry measure of reactive changes in arterial tone. However, intra- and inter-participant variability in RHI was high, and about half had baseline values corresponding to normal endothelial function. In contrast, praliciguat treatment was associated with reduction in plasma ADMA and increase in the l-arginine/ADMA ratio. ADMA competitively inhibits the generation of NO from l-arginine by NO synthases. This, in turn, can reduce the bioavailability of NO and lead to endothelial dysfunction [21, 22]. Plasma ADMA is elevated in diabetes, hypertension and diabetic nephropathy, and is an independent risk factor for all-cause mortality and cardiovascular disease [23–25]. Thus, modulation of ADMA levels and l-arginine/ADMA ratio by praliciguat may imply an impact on endothelial function which, if sustained, could be associated with cardiovascular and renal benefit.

The results from this trial support an extensive body of non-clinical evidence demonstrating that praliciguat and sGC stimulators lower BP, improve metabolic homeostasis by reducing blood glucose, increase insulin sensitivity, and reduce serum lipids [7, 26]. Potential mechanisms for a positive metabolic effect of sGC stimulation could include promoting insulin access to tissues, enhancing insulin receptor signalling and/or improving mitochondrial function [26]. Non-clinical investigations have suggested an interdependence between insulin and the NO–sGC–cGMP signalling pathway in both the endothelium and metabolically active tissues that are disrupted in the metabolic syndrome [27, 28].

Reduction in total cholesterol, LDL-cholesterol and triacylglycerols could be mediated by enhanced insulin receptor signalling and/or improved function of proteins such as peroxisome proliferator-activated receptors (PPARs), key regulators of lipid and carbohydrate metabolism [29]. Dietary nitrates, which also increase plasma cGMP in humans [30], have been shown to increase metabolism of fatty acids in skeletal muscle through an NO/sGC/cGMP/PPAR-mediated mechanism in rats [29–31].

The pharmacokinetic profile of praliciguat in individuals with diabetes and hypertension was found to be consistent with that observed in healthy volunteers, including rapid absorption, a high volume of distribution suggesting extensive dispersal into tissues, and a *t*_½_ supportive of once daily dosing [10]. The large volume of distribution is consistent with high tissue-to-plasma concentration ratios observed in animal studies of praliciguat [8, 9]. High local drug concentrations can produce sustained pharmacological effects [32] and supports investigation in conditions associated with impaired tissue NO–sGC–cGMP signalling.

Overall, praliciguat was well tolerated in this study. Similar to reports from clinical trials of other sGC stimulators [33, 34] and consistent with the known pharmacological effects of drugs that modulate NO signalling, headache and gastrointestinal symptoms were among the most frequently observed TEAEs. These TEAEs were mild, transient and subsided with continued therapy. Headaches may be related to the BP-lowering effects of praliciguat; however, headaches in this trial occurred at a similar rate in placebo-treated participants. Among praliciguat-treated participants experiencing headache, most occurred in the first week in those who started on 40 mg once daily dosing, suggesting both that this adverse event may be regimen-related and that 1 week of 20 mg twice daily dosing may have reduced the likelihood of headache when the dose was changed to 40 mg once daily in the second week. Gastrointestinal TEAEs, including nausea, diarrhoea, abdominal pain and dyspepsia, were the only types of event that were clearly imbalanced between praliciguat- and placebo-treated participants. sGC is present in smooth muscle cells, including in the gastrointestinal tract, and NO signalling plays a role in gastrointestinal physiological functioning, including gastrointestinal-tract motility [35, 36]. A similar pattern of gastrointestinal adverse effects is noted in the prescribing information for the only marketed sGC stimulator, riociguat (Adempas) [37].

Hypoglycaemia was reported with equal frequency in praliciguat- and placebo-treated participants, and, with the exception of a single placebo-treated individual, was observed exclusively in those receiving concomitant insulin therapy. This suggests that insulin and/or characteristics of individuals requiring insulin may have played a role in these events. Supporting this interpretation are results from another small trial of praliciguat in individuals with diabetes and hypertension, in which hypoglycaemia was reported in only one person on concomitant insulin, and other trials conducted in healthy volunteers where hypoglycaemia was not observed [10].

A single SAE occurred in this trial: an upper gastrointestinal haemorrhage event in a participant without a history of gastrointestinal bleeding. The prescribing information for riociguat contains a warning for bleeding because a disproportionate number of serious bleeding events were observed in Phase III trials [33, 34, 37] in individuals with pulmonary hypertension. Because sGC is expressed in platelets and NO–sGC–cGMP signalling has been reported to inhibit platelet activation [38], we assessed platelet function in this trial using multiple methods. Praliciguat treatment did not show an effect on platelet function, consistent with the results of prior clinical studies of praliciguat in healthy volunteers [10]. It is not known whether the large tablet burden required in this trial may have contributed to the SAE. A potential impact of praliciguat on platelet function and/or risk of serious bleeding events warrants continued clinical vigilance.

Several limitations of this study need to be noted. The trial was not powered for inference testing for any of the outcomes examined, so that all results must be considered hypothesis-generating, requiring future confirmation. The trial examined only 26 individuals and the treatment duration of 14 days was too short to observe meaningful changes in long-term glycaemic control (e.g. HbA_1c_ levels) or to evaluate the durability of effects. The in-clinic treatment phase added lifestyle modification and imposed medication adherence as potential factors influencing the results. In addition, the number of placebo-treated participants was small, resulting in wide CIs for praliciguat–placebo treatment difference comparisons. Finally, trial entry criteria were liberal, allowing enrolment of individuals with type 2 diabetes and hypertension independent of treatment regimen.

The promising results observed in this study, including positive trends in metabolic and haemodynamic variables, support further clinical investigation of praliciguat. Praliciguat is being evaluated in separate Phase II trials in individuals with diabetic nephropathy and heart failure with preserved ejection fraction.

## Electronic supplementary material

ESM (PDF 82.9 kb)

## Data Availability

The datasets generated during and/or analysed during the current study are not publicly available due to their proprietary nature but are available from the corresponding author on reasonable request.

## Abbreviations

ABPM: Ambulatory BP monitoring
ACEi: ACE inhibitor
ADMA: Asymmetric dimethylarginine
AI_75_: Augmentation index corrected to a heart rate of 75 bpm
ARB: Angiotensin receptor blocking agent
ARU: Aspirin reaction units
bpm: Beats/min
cGMP: Cyclic GMP
C_max_: Maximum observed plasma concentration
DBP: Diastolic BP
LS: Least squares
MAP: Mean arterial pressure
PPAR: Peroxisome proliferator-activated receptor
PRU: P2Y_12_ reaction units
RHI: Reactive hyperaemia index
SAE: Serious adverse event
SBP: Systolic BP
sGC: Soluble guanylate cyclase
TEAE: Treatment-emergent adverse event
T_max_: Time of C_max_
TRAP: Thrombin receptor activating protein
V_z_/F: Apparent volume of distribution during the terminal elimination phase
