# Supplementary material for: An exploratory, randomised, placebo-controlled, 14 day trial of the soluble guanylate cyclase stimulator praliciguat in participants with type 2 diabetes and hypertension
Source: Diabetologia. 2019 Dec 19;63(4):733–43. doi: 10.1007/s00125-019-05062-x (PMC7054374; doi:10.1007/s00125-019-05062-x)
Supplement: Supplementary file 1 — (PDF 82.9 kb) [file 125_2019_5062_MOESM1_ESM.pdf]

Supplemental Table 1. Praliciguat PK On Dosing Day 7 and Day 14 (Mean [SD])

Dosing Day	BID/QD (N=10)	QD/QD (N=10)	
**Day 7**	**20 mg BID**	**40 mg QD**	
T_{max} [a] (h)	1.0 (1.0, 3.0)	1.0 (1.0, 6.0)	
C_{max} (ng/mL)	117 (33)	242 (80)	
C_{max}/ Dose (ng/mL/mg)	5.84 (1.63)	6.05 (2.00)	
AUC_{tau} (h·ng/mL)	795 (186)	2200 (591)	
AUC_{tau}/ Dose (h·ng/mL/mg)	39.7 (9.3)	54.9 (14.8)	
C_{trough} (ng/mL)	52.4 (12.8)	57.2 (14.9)	
RC_{max}	1.69 (0.72)	1.66 (0.91)	
$RAUC_{tau}$	2.22 (0.25)	1.96 (0.38)	
RC_{trough}	2.76 (0.68)	3.77 (0.45)	
**Day 14**	**40 mg QD**	**40 mg QD**	**Overall (N=20)**
T_{max} [a] (h)	1.0 (1.0, 3.0)	1.0 (1.0, 6.0)	1.0 (1.0, 6.0)
C_{max} (ng/mL)	181 (47)	250 (139)	217 (109)
C_{max}/ Dose (ng/mL/mg)	4.53 (1.17)	6.25 (3.48)	5.44 (2.73)
AUC_{tau} (h·ng/mL)	2080 (442)	2910 (770)	2520 (750)
AUC_{tau}/ Dose (h·ng/mL/mg)	52.1 (11.0)	72.7 (19.2)	62.9 (18.7)
AUC_{last} [b] (h·ng/mL)	17100 (6980)	23600 (8950)	20500 (8530)
C_{trough} (ng/mL)	65.7 (23.0)	90.7 (26.2)	78.9 (27.3)
RC_{max}	-	1.55 (0.75)	-
$RAUC_{24}$ [c]	3.29 (0.80)	2.60 (0.54)	2.93 (0.74)
RC_{trough} [d]	3.45 (1.54)	6.02 (1.27)	4.74 (1.90)
CL/F (L/h)	20.0 (4.3)	14.7 (3.9)	17.2 (4.8)
V_z/F (L)	5570 (1530)	3880 (1370)	4680 (1650)
Terminal $t_{1/2}$ (h)	197 (57.4)	183 (43)	190 (50)
Effective $t_{1/2}$ (h)	45.8 (13.5)	34.2 (9.1)	39.7 (12.6)

%CV=intersubject variability [percent]; AUC_{tau}=area under the plasma concentration-time curve during a dosing interval; CL/F=apparent total body clearance after oral administration; C_{max}=maximum observed plasma concentration, occurring at T_{max}; C_{trough}=plasma concentration observed at the end of a dosing interval (collected before the next administration); Effective $t_{1/2}$= one-compartment elimination rate that would lead to the observed degree of AUC accumulation; $RAUC_{24}$=accumulation ratio calculated from 24-hour AUC on Day 14 relative to Day 1; $RAUC_{tau}$=accumulation ratio calculated from AUC_{tau} on Day 7 relative to Day 1; RC_{max}=accumulation ratio calculated from C_{max} on Day 14 relative to Day 1; RC_{trough}=accumulation ratio calculated from C_{trough} on Day 14 relative to Day 1; Terminal $t_{1/2}$=apparent terminal elimination phase half-life; T_{max}=time of maximum observed plasma concentration; V_z/F=apparent volume of distribution during the terminal phase.

a Median (minimum, maximum)

b T_{last} = 38 days post-final dose (Day 42)

c Day 14 AUC_{tau} / Day 1 AUC_{tau} for QD/QD regimen; Day 14 AUC_{tau} / Day 1 AUC_{24} for BID/QD regimen

d Day 14 24-hour postdose concentration / Day 2 morning predose concentration

Supplemental Table 2. Biomarker Assessments

Biomarker	Placebo (N=6)		Praliciguat (N=20)		LS mean difference between praliciguat and placebo (95% CI)
	Baseline	LS mean change (95% CI)	Baseline	LS mean change (95% CI)	
Cyclic guanosine monophosphate (ng/mL)	0.82 ± 0.26	0.09 (-0.19,0.38)	0.93 ± 0.36	0.33 (0.18, 0.49)	0.24 (-0.08, 0.56)
Arginine (µg/mL)	12.52 ± 3.94	0.04 (-1.19, 1.27)	13.23 ± 2.82	0.82 (0.15, 1.49)	0.78 (-0.63, 2.18)
Asymmetric dimethylarginine (ng/mL)	115.10 ± 18.20	1.97 (-4.97, 8.91)	104.94 ± 9.03	-8.68 (-12.34, -5.02)	-10.65 (-18.68, -2.61)
Arginine/asymmetric dimethylarginine ratio[a]	107.67 ± 24.02	-1.99 (-14.67, 10.69)	125.77 ± 23.16	20.17 (13.42, 26.91)	22.16 (7.52, 36.79)
Lipoprotein (a) (µg/mL)	291.4 ± 172.0	80.6 (-4.9, 166.1)	257.6 ± 269.8	62.0 (18.2, 105.9)	-18.6 (-114.7, 77.6)
Apolipoprotein B (µg/mL)	1147 ± 346.4	-50.5 (-204.1,103.0)	1012.2 ± 274.1	-169.5 (-252.7, -86.4)	-119.0 (-294.9, 56.9)
Apolipoprotein C3 (µg/mL)	451.8 ± 188.0	-104.3 (-158.6, -50.0)	417.1 ± 186.9	-105.0 (-134.7, -75.3)	-0.8 (-62.7, 61.2)
Soluble intercellular adhesion molecule 1 (µg/mL)	0.57 ± 0.27	0.03 (-0.07,0.14)	0.39 ± 0.09	-0.03 (-0.09, 0.02)	-0.07 (-0.19, 0.06)
Soluble vascular cell adhesion molecule 1 (µg/ml)	0.64 ± 0.26	0.03 (-0.09,0.15)	0.56 ± 0.16	-0.02 (-0.08, 0.05)	-0.04 (-0.18, 0.09)
Interleukin 6 (pg/ml)[b]	1.61 ± 0.70	0.18 (-0.72, 1.08)	1.40 ± 0.73	0.67 (0.12,1.23)	0.49 (-0.57, 1.56)
Interleukin 8 (pg/ml)	6.55 ± 3.20	0.75 (-1.86, 3.36)	12.11 ± 29.12	0.49 (-0.93, 1.92)	-0.26 (-3.24, 2.73)
Tumor necrosis factor alpha (pg/ml)	4.11 ± 1.67	-0.06 (-0.72, 0.61)	3.60 ± 1.18	-0.06 (-0.42, 0.30)	-0.004 (-0.76, 0.75)
Interferon gamma (pg/ml)	15.15 ± 21.95	-0.22 (-3.85, 3.41)	4.91 ± 2.58	-0.39 (-2.34, 1.56)	-0.17 (-4.41, 4.07)
Serum amyloid A (µg/ml)[c]	19.75 ± 23.38	1.56 (0.78, 3.11)	9.36 ± 7.84	1.04 (0.72, 1.51)	0.67 (0.30, 1.48)
C-reactive protein (µg/ml)[c]	7.26 ± 6.57	1.22 (0.57, 2.60)	3.52 ± 3.30	1.35 (0.90, 2.03)	1.11 (0.46, 2.64)

[a]Arginine/asymmetric dimethylarginine ratio was calculated post hoc by first converting arginine in µg/ml to ng/ml; [b]Interleukin-6 was detectable in 7 out of 20 patients in the treatment group and 5 out of 6 patients in the placebo group. No imputations were performed; [c]C-reactive protein and serum amyloid A were log-transformed prior to analysis, LS mean values were exponentiated back to original scale
